# Prediction of Parkinson Disease Using Long-Term, Short-Term Acoustic Features Based on Machine Learning

**DOI:** 10.3390/brainsci15070739

**Published:** 2025-07-10

**Authors:** Mehdi Rashidi, Serena Arima, Andrea Claudio Stetco, Chiara Coppola, Debora Musarò, Marco Greco, Marina Damato, Filomena My, Angela Lupo, Marta Lorenzo, Antonio Danieli, Giuseppe Maruccio, Alberto Argentiero, Andrea Buccoliero, Marcello Dorian Donzella, Michele Maffia

**Affiliations:** 1 Department of Mathematics and Physics “E. De Giorgi”, University of Salento, Via Lecce—Arnesano, 73100 Lecce, Italy; mehdi.rashidi@unisalento.it (M.R.); chiara.coppola@unisalento.it (C.C.); giuseppe.maruccio@unisalento.it (G.M.); 2 Department of Human and Social Sciences, University of Salento, 73100 Lecce, Italy; serena.arima@unisalento.it; 3 Department of Biological and Environmental Science and Technology, University of Salento, Via Lecce—Monteroni, 73100 Lecce, Italy; andrea-claudio.stetco@studenti.unisalento.it; 4 Department of Experimental Medicine, University of Salento, Via Lecce—Monteroni, 73100 Lecce, Italy; debora.musaro@unisalento.it (D.M.); marco.greco@unisalento.it (M.G.); marina.damato@unisalento.it (M.D.); antonio.danieli@unisalento.it (A.D.); 5 Division of Neurology, Vito Fazzi Hospital, 73100 Lecce, Italy; filomenamy28@gmail.com (F.M.); angela87lupo@gmail.com (A.L.); martatnfp@gmail.com (M.L.); 6 Institute of Nanotechnology, CNR—Nanotec and INFN Sezione di i Lecce, via-ia Per Monteroni, 73100 Lecce, Italy; 7 Department of Medicine and Surgery, University of Parma, 43121 Parma, Italy; alberto.argentiero@unisalento.it; 8 Department of Research and Development (R&D), GPI S.p.A., 38123 Trento, Italymarcello.donzella@gpi.it (M.D.D.); 9 Human Science Department, University of Verona, 37129 Verona, Italy

**Keywords:** Parkinson’s disease, machine learning, vocal features, mel-frequency cepstral coefficient

## Abstract

**Background:** Parkinson’s disease (PD) is the second most common neurodegenerative disorder after Alzheimer’s disease, affecting countless individuals worldwide. PD is characterized by the onset of a marked motor symptomatology in association with several non-motor manifestations. The clinical phase of the disease is usually preceded by a long prodromal phase, devoid of overt motor symptomatology but often showing some conditions such as sleep disturbance, constipation, anosmia, and phonatory changes. To date, speech analysis appears to be a promising digital biomarker to anticipate even 10 years before the onset of clinical PD, as well serving as a useful prognostic tool for patient follow-up. That is why, the voice can be nominated as the non-invasive method to detect PD from healthy subjects (HS). **Methods:** Our study was based on cross-sectional study to analysis voice impairment. A dataset comprising 81 voice samples (41 from healthy individuals and 40 from PD patients) was utilized to train and evaluate common machine learning (ML) models using various types of features, including long-term (jitter, shimmer, and cepstral peak prominence (CPP)), short-term features (Mel-frequency cepstral coefficient (MFCC)), and non-standard measurements (pitch period entropy (PPE) and recurrence period density entropy (RPDE)). The study adopted multiple machine learning (ML) algorithms, including random forest (RF), K-nearest neighbors (KNN), decision tree (DT), naïve Bayes (NB), support vector machines (SVM), and logistic regression (LR). Cross-validation technique was applied to ensure the reliability of performance metrics on train and test subsets. These metrics (accuracy, recall, and precision), help determine the most effective models for distinguishing PD from healthy subjects. **Result:** Among all the algorithms used in this research, random forest (RF) was the best-performing model, achieving an accuracy of 82.72% with a ROC-AUC score of 89.65%. Although other models, such as support vector machine (SVM), could be considered with an accuracy of 75.29% and a ROC-AUC score of 82.63%, RF was by far the best one when evaluated across all metrics. The K-nearest neighbor (KNN) and decision tree (DT) performed the worst. Notably, by combining a comprehensive set of long-term, short-term, and non-standard acoustic features, unlike previous studies that typically focused on only a subset, our study achieved higher predictive performance, offering a more robust model for early PD detection. **Conclusions:** This study highlights the potential of combining advanced acoustic analysis with ML algorithms to develop non-invasive and reliable tools for early PD detection, offering substantial benefits for the healthcare sector.

## 1. Introduction

### 1.1. Background

It goes without saying that Parkinson’s disease (PD) is the second most prevalent Neurodegenerative disorder after Alzheimer’s disease [1]. The incidence of Parkinson’s has risen yearly, and this trend is estimated to increase the number of PD patients to 17 million cases by 2040 [2]. This issue can give rise to increasing socioeconomic burdens worldwide [3]. People with Parkinson’s disease (PWP) are characterized by motor symptoms, including tremor, bradykinesia, rigidity, and non-motor symptoms such as REM sleep behavior disorder (RBD), voice impairment, depression, and anxiety [4,5,6].

The cause of those symptoms is neuronal damage caused by the accumulation of alpha-synuclein protein in a brain region called substantia nigra [7]. Unfortunately, among all motor and non-motor symptoms, some of them can be manifested prior to neurons loss at the prodromal phase, including speech, REM sleep behavior disorder (RBD), and hyposmia [8,9,10]. In the context of the early diagnosis of Parkinson’s disease (PD), speech biomarkers offer a non-invasive and low-cost alternative to other expensive [11] and cumbersome diagnostic methods to detect early symptoms of PD such as REM sleep behavior disorder (RBD), hyposmia (reduced sense of smell), and neuroimaging. RBD, which manifests as abnormal behaviors during REM sleep, is considered by many to be an early indication of synucleinopathies, including PD [12]. Studies have shown that individuals with isolated RBD have a high risk of rapidly developing PD in the future. However, not all patients with RBD will progress to PD, and the time frame for such progression can vary [13]. Hyposmia is also a common early symptom of PD, often experienced by patients years before physiological symptoms such as motor impairments begin [14]. Neuroimaging techniques, such as dopamine transporter DaTscan and MRI, represent significant advancements in detecting brain changes associated with Parkinson’s disease. However, the cost of these tests is high and they are not universally available. Moreover, they may sometimes reveal coexistent findings with other neurodegenerative diseases, which can reduce diagnostic accuracy [15]. Fundamentally, speech biomarkers provide a practical and inexpensive method for the early detection of Parkinson’s disease.

Hypokinetic dysarthria, a distinctive speech disorder associated with PD, is characterized by a range of vocal difficulties, including diminished vocal power, a monotonous pitch range, and impaired articulation [16]. These impairments decrease interaction with people, leading to social isolation and psychological distress, such as depression [17]. In recent years, scientists have found out that more than 90 percent of PD patients suffer from vocal impairments [18] and that the voice can reveal PD up to 10 years before the manifestation of motor symptoms. This indicates that the voice can be one of the best digital biomarkers to predict Parkinson’s disease at an early stage [19]. Apart from using voice as a digital biomarker to detect Parkinson’s disease at an early stage, speech can also be influenced by other motor symptoms. For instance, patients with the postural instability/gait difficulty (PIGD) motor subtype show more pronounced speech impairments compared to those with the tremor-dominant subtype. Individuals with PIGD experience severe disruptions in both speech timing and gait, suggesting potential shared underlying mechanisms that affect both speech and gait [20]. Utilizing disease severity scales, such as Hoehn and Yahr staging and MDS-UPDRS scores, provides a deeper understanding of how disease progression impacts speech. For example, MDS-UPDRS scores generally increase by over 30% with each stage progression on the Hoehn and Yahr scale, and with each 5-year increase in disease duration over the first 15 years. These changes reflect the worsening of both motor and non-motor symptoms and offer valuable insights into disease progression [21]. Nonetheless, longitudinal approach can be carried out based on UPDRS, while a cross-sectional approach can use voice recordings at one specific point in time.

In this era, thanks to the remarkable technological revolution, artificial intelligence (AI) and machine learning (ML) can be adopted to analyze voice signals as digital biomarkers to provide non-invasive tools that increase the possibility of monitoring and analyzing signals to predict PD without adopting interventional procedures [22]. Machine learning models are integrated in smartphone applications to differentiate Parkinson’s disease patients from controls without the need for invasive measurements. These applications can also be used by neurologists to monitor patients, highlighting the significant role of AI and machine learning in telemedicine [23].

Moreover, ML models will result in high accuracy with minimum human error [24]. Feature extraction is a fundamental step in machine learning, especially when preparing data for training models. In the context of Parkinson’s disease (PD), identifying and including features that are strongly linked to the condition is essential for accurate detection. The inclusion of more relevant and informative features can greatly improve the effectiveness of machine learning algorithms. Both long-term and short-term features can be employed [25]. Moreover, according to reliable research, pitch period entropy (PPE) and recurrence period density entropy (RPDE) could be considered suitable inputs for machine learning models [26]. Long-term features include fundamental frequency (F0) (pitch), formant frequencies, jitter, and smoothed cepstral peak prominence (CPPS) [27,28], while short-term features consist of Mel-frequency cepstral coefficients (MFCCs). Due to their high inter-feature correlation, classification accuracy has been reduced when utilizing long term features in comparison to short term feature [29]. Among these features, MFCCs, specifically MFCC2, plays a significant role in interpretation and classification [30]. Classification approach increases the opportunity to predict disease by utilizing vocal features [31]. These models include random forest (RF), logistic regression (LR), naïve Bayes (NB), decision tree (DT), K-nearest neighbors (KNN), and support vector machines (SVM) which are the most common ML models employed in medical research [24]. Nowadays, overfitting can occur when scientists try to model development, so after building models, unseen data cannot be detected by them, that is why cross-validation is one of the resampling approaches to build models, leading to reduced overfitting and increasing robustness of model [32]. Finally, all models can leverage vocal features to accurately classify individuals with Parkinson’s disease (PD) from healthy subjects (HS) [33]. Once all algorithms have been implemented, several metrics have been obtained such as accuracy, recall score, precision [34], ROC-AUC score [35], and F1-score [36] to report performance of models, demonstrating which models perform better at predicting Parkinson’s disease (PD) from healthy subjects (HS) [37].

In this study, we aim to develop and compare a suite of machine learning models for the early, non-invasive detection of Parkinson’s disease based solely on vocal biomarkers. To this end, we extract a comprehensive battery of long-term (fundamental frequency, formants, jitter, and CPPS), short-term (MFCCs), and non-standard (PPE and RPDE) speech features and train six well-established classifiers (RF, LR, NB, DT, KNN, and SVM) under a k-fold cross-validation scheme. Model performance is evaluated using accuracy, recall, precision, F1-score, and ROC AUC, and feature importance analyses are conducted to identify the most discriminative vocal parameters. The remainder of this manuscript is organized as follows. In Section 2, we describe the dataset and our feature extraction pipeline. Section 3 details the machine learning methods and validation framework based on their metrics and compares the experimental results, and Section 4 discusses the clinical and telemedicine implications. Section 5 addresses the limitations and outlines future directions, and Section 6 concludes the paper with a summary of our key findings.

### 1.2. Related Work

Several studies have explored the use of machine learning (ML) to predict Parkinson’s disease at an early stage. We also focus on developing algorithms to predict PD more effectively than those used in previous studies.

Several research works have been conducted on the prediction of Parkinson’s disease at the earliest stage with the assistance of machine learning (ML). In this study, we also emphasize the development of algorithms to predict PD more accurately than those already implemented.

Wroge et al. (2018) [38] elaborated on the potential of voice-derived biomarkers and machine learning-based diagnosis for Parkinson’s disease. They evaluated different classification models such as decision trees, random forests, support vector machines, and artificial neural networks using 10-fold cross-validation. Results emphasized that deep learning approaches were more precise than traditional methods. The study also evaluated different sets of features and data segmentation methods. Altogether, their findings confirm voice analysis as a valuable and scalable means of early PD detection.

Iyer et al. (2023) [39] applied both traditional machine learning algorithms (i.e., random forest (RF) and logistic regression (LR)) and deep models based on Convolutional Neural Networks (CNN). While they experimented with features like MFCC and cepstral coefficients in their models, the traditional machine learning models were not superior to the CNN model. That is, the deep learning model outperformed the traditional classifiers, with the former proving to be more accurate and stable in classifying Parkinson’s disease based on voice features.

Max Little (2009) [26] aimed to introduce new measures of dysphonia such as pitch period entropy (PPE) and achieved high accuracies utilizing SVM, thus demonstrating their potential for telemonitoring. Non-standard features were addressed in this research. Although they also employed long-term features along with non-standard features, they did not explore MFCC as the most significant feature for diagnosing Parkinson’s disease from healthy individuals.

Suppa et al. (2022) [40] reported that voice abnormalities occurred even during the early stages of Parkinson’s disease (PD) and became more pronounced as the disease progressed. This was supported by high sensitivity in discriminating between normal subjects and PD patients at both early and mid-advanced stages. While L-Dopa therapy produced some improvement in voice quality, it did not fully restore it, as shown by fluctuations in voice features between OFF and ON medication states. Interestingly, this study introduced a new machine learning—a learned score that, for the first time, showed significant correlations with clinical ratings and voice features.

## 2. Materials and Methods

**Figure 1:**
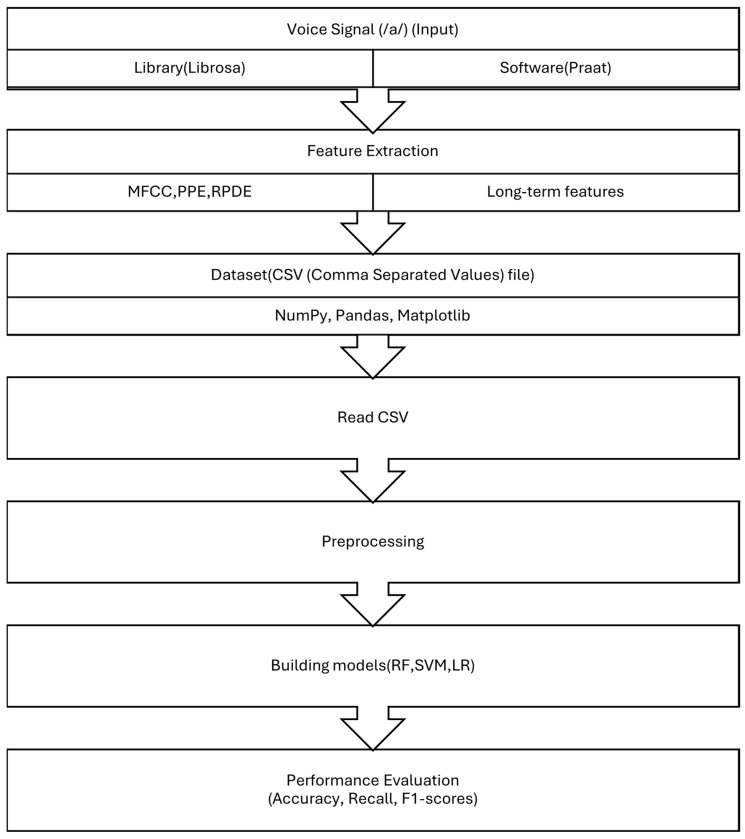
Typical pipeline for voice-based analysis.

The methodology of this study was designed as a cross-sectional analysis and was implemented in five main steps, as illustrated in Figure 1. Initially, voice recordings were obtained from a publicly available dataset hosted on Figshare [39,41,42]. These recordings, which contained sustained vowel phonations (/a/), were converted into numerical representations.

In the second step, acoustic features were extracted. We used Praat software (version 6.4 in 2024) to extract long-term features (shimmer, jitter, and HNR) and the Librosa library in Python for short-term and non-standard features (MFCC, PPE, and RPDE). These features were combined to construct a comprehensive dataset which was saved as a CSV (Comma-Separated Values) file.

Next, using Python and relevant libraries such as NumPy, Pandas, and Matplotlib, the dataset was read and visualized. Preprocessing and normalization were carried out using Scikit-learn. A train–test split and 5-fold cross-validation were applied to ensure the generalizability of our models and to mitigate overfitting.

In the fourth stage, we built classification models using six machine learning algorithms, including random forest (RF), support vector machine (SVM), and logistic regression (LR). Finally, the fifth step involved evaluating model performance using metrics such as accuracy, recall, and F1-score to determine the most effective algorithm for Parkinson’s disease detection.

### 2.1. Dataset Preparation

The dataset was constructed by converting WAV-format voice recordings into numerical features. The audio signals were sampled at 8 kHz with 16-bit resolution. Prior to feature extraction, the recordings were denoised using Audacity software (version 3.7.0). Amplitude-based filtering was applied to exclude irrelevant signal segments, with threshold ranges set between 75 dB and 300 dB for male speakers and between 100 dB and 600 dB for female speakers.

Although the raw voice recordings used in this work were collected by Iyer et al. [41] from a publicly available repository, we applied custom features extraction including Jitter, shimmer, MFCC, and CPPS using Praat software and Librosa on python. A total of 41 healthy voices (16 males, 25 females) and 40 voices from PD patients (21 males, 19 females) were studied. All the PD patients which were enrolled had a mean age of 66.6 ± 9.0 years, whereas in the healthy controls, the mean age was 47.9 ± 14.5 years. The Hoehn & Yahr staging scale was 2.1 ± 0.4 for Parkinson patients. The voices were prepared for feature extraction [39]. All participants pronounced the sustained vowel /a/. To select the appropriate vocal test, different approaches were considered, such as running speech. However, sustained vowel tests were preferred as they can better induce speech difficulties. Even though running speech is more realistic than sustained vowel tests, it is more difficult to analyze [25,39].

### 2.2. Acoustic Signal Features

Although features can be extracted using software such as Praat [43] and the Parselmouth library in Python, this study used Praat (the original software) to extract long-term features (predictors), including jitter, shimmer, and smoothed cepstral peak prominence (CPPS). Notably, The Mel-frequency cepstral coefficient (MFCC), pitch period entropy (PPE), and recurrence period density entropy (RPDE) were extracted using the librosa in python.

Signals of voices in WAV format were initially pre-processed with Audacity to remove noise and normalize amplitudes of signals between −1 and 1. Additionally, the silence parts in the beginning and the end were cut out. For the acoustic part of feature extraction, we have employed Praat and Librosa libraries. In Praat, the pitch was set within the range of 75–600 Hz, the intensity value was limited to the interval 50–100 dB, the frequency of the spectrogram was from 0 to 5000 Hz, and for formants the analysis was performed with a maximum frequency of 5500 Hz only.

Based on the pitch data obtained, pitch period entropy (PPE) was formed by converting them into semitones and calculating the entropy. The recurrence period density entropy (RPDE) was calculated with the pyrpde package by phi parameters: data_float32, tau = 30, dim = 4, epsilon = 0.01, and t-max = 1500. Librosa was used to provide Mel-Frequency Cepstral Coefficients (MFCCs) as a representation of sound. It extracted 12 values per file and calculated their means. All the data extracted were then put together into a format compatible for training the model. As compared to the works of others who use pre-tabulated data in excel, our dataset was generated from scratch using voice files through the pipeline given above.

#### 2.2.1. Long Term Features

Long-term features consist of various types of parameters: fundamental frequency (F0) or pitch, which represents the rate at which the vocal folds vibrate; jitter, which measures variations in F0 across vocal cycles; shimmer, indicating fluctuations in amplitude between cycles [25,44]; and cepstral peak prominence (CPP), which represents the difference between the maximum amplitude and noise [8,28]. Formants, which are the spectral peaks of the speech spectrum, correspond to the resonant frequencies of the vocal tract [45]. All these features can be used to train our model as input data to assist decision making and predict outcome (automatic Parkinson’s disease detection based on the combination of long-term acoustic features and Mel frequency cepstral coefficients (MFCC)). These acoustic signal features should be used for automatic detection of Parkinson’s disease. These features can characterize signal properties over extended periods, including amplitude variations (shimmer) and frequency fluctuations (jitter).

#### 2.2.2. Pitch Period Entropy (PPE)

The PPE measures irregularities in speech pitch, which is relevant for analyzing speech impairments and Parkinson’s disease (PD) since voice control may be impaired. This parameter evaluates the difference between natural variations (such as vibrato and microtremor) and pathological speech associated with PD. In fact, this factor shows how the human ear can perceive pitch change. If the entropy is computed (a measure of randomness or uncertainty) of pitch variations, PPE can provide reliable measurement to detect PD. This feature can be useful when our study encounters uncontrollable confounding effects including noisy and normal acoustic environments, and also improve the classification of healthy Parkinson patients [25].

#### 2.2.3. The Recurrence Period Density Entropy (RPDE)

The recurrence period density entropy (RPDE) can be used to evaluate regularity or periodicity of a voice signal. For example, in healthy individuals, the vocal folds produce voice consistently and follow a regular pattern. On the other hand, voice disorders such as Parkinson’s disrupt this regular pattern, leading to unstable vibrations or irregularities, resulting in a less steady voice. RPDE analyzes the recurrence of similar patterns within the signal over time. RPDE is normalized between 0 and 1. Values closer to 0 indicate higher regularity, while values closer to 1 represent greater irregularities in the voice signal. This parameter can identify general voice disorders and the steady vibration of the vocal folds [26,46].

#### 2.2.4. Short Term Feature (Mel Frequency Cepstral Coefficients)

Mel-frequency cepstral coefficients (MFCC) were first introduced for automatic speech recognition systems in 1980 [47]. Over time, this feature was applied to detect voice disorders and is now widely used as a reliable feature for detecting PD [48]. This short-term feature has non-correlation with some other features, particularly long-term features, making it a reliable factor in improving classification accuracy. Negative MFCC values indicate a higher concentration of energy in the higher frequency bands of the Mel filter. Parkinson’s disease often manifests in voice changes, including hoarseness and breathiness, which are characterized by increased high-frequency energy and, consequently, more negative MFCC values [29]. Table 1 showed a list of features used as input data in the dataset:

Preprocessing plays a crucial role in machine learning, as the proper execution of this stage ensures that models perform more reliably. First, the dataset was read using the Pandas’ library in Python. Then, the data in the table was converted into a matrix array. All numerical values were standardized and split into 80% training data and 20% test data. Additionally, K-fold cross-validation was applied to enhance the validation of models on both the training datasets.

### 2.3. Classification Algorithms

In recent times, various types of models have been employed for detecting Parkinson’s disease (PD) from healthy subjects (HS). This research utilized six common algorithms. To improve the efficiency of a model, fine-tuning of hyperparameters was performed in various ways using grid search. The hyperparameters determining the random forest classifier are the number of trees (n_estimators), the maximum depth of each tree (max_depth), the minimum number of samples required to split an internal node (min_samples_split), and the minimum number of samples required to be at a leaf node (min_samples_leaf). Logistic regression utilized hyperparameters dealing with the regularization strength (C), the penalty type (l1 or l2), and the optimization solver (liblinear). For the support vector machine (SVM), the kernel type (kernel), regularization (C), and gamma were values that have been changed. In a similar manner, for naïve Bayes, var_smoothing was the element to make sure that the operations did not cause any noise. In the K-nearest neighbors (KNN) model, hyperparameters such as the number of neighbors (n_neighbors) and the search algorithm (algorithm) were tuned.

#### 2.3.1. Random Forest (RF)

Random forest is a popular ensemble learning method used in machine learning and data science. It works by creating multiple decision trees on different subsets of the training data. These individual trees are then combined to make a final prediction, often through majority voting or averaging. This approach helps to reduce overfitting and improve the overall accuracy and reliability of the model compared to a single decision tree [49].

#### 2.3.2. Logistic Regression (LR)

The LR is one of the popular statistical-based models employed to solve classification issues in ML by probabilities that should be regarded in biological research particularly when ML algorithms are implemented where the target variable is categorical. As mentioned before, this dataset contains one column labeled as the target for detecting PD. Logistic regression assumes a linear relationship between independent and dependent variables, which can limit its applicability in complex, nonlinear relationships. This model calculates probabilities based on sigmoid function between the range of [0, 1] as shown in the equation below [24].

$$\sigma (z)=\frac{1}{1+e^{-z}}$$

#### 2.3.3. Naive Bayes (NB)

Naive Bayes (NB) is one of the simplest algorithms adopted by machine learning models. This model is applied as supervised learning based on conditional probability. It is much faster than other supervised learning models because it solely calculates probability and has high accuracy with categorical features. However, Naive Bayes has a drawback, that is, if it encounters unseen data in the training input, it may be unable to make predictions, leading to a probability of 0 [50].

#### 2.3.4. Decision Tree

A Decision tree is acommon ML model in classification. Each step shows a feature of the data, while the branch is related to the value of that feature. By considering both factors, the model can classify the data points into a specific category. This model is interpreted by humans easily because of its tree structure. It is also useful in handling missing data. However, overfitting, sensitivity to noise, and bias toward the majority class are the disadvantages of the models [31].

#### 2.3.5. K-Nearest Neighbor (KNN Classifier)

Euclidean distance function is used to compute data and classify new data points based on similar measures on the KNN model or lazy learning. On this model, the classification of a data point is determined by the majority vote of its nearest neighbors. The K-NN algorithm stores all available data points and classifies new data points based on their similarity to existing ones. This means that new data can be easily categorized by comparing them with the most similar existing data points. The most challenging part of these models is selecting the optimal number of neighbors [37].

#### 2.3.6. Support Vector Machine (SVM)

The SVM uses a hyperplane to optimally separate two classes, maximizing the margin between them. In this case, the classes are HS and PD. This hyperplane serves as a decision boundary, which is identified by the SVM algorithm. The decision boundary divides the data space into two distinct regions, i.e., normal and PD. The geometric margin refers to the distance from the decision boundary to the nearest data point. When the data is linearly separable and the resolution limits are clearly separated by the hyperplane, the geometric margin is positive. The objective is to find a hyperplane that maximizes this margin. When the training data are linearly separable, a single linear decision boundary exists, separating the normal data above the hyperplane from the PD data below it [51,52].

### 2.4. Cross Validation

Cross validation is one of the most popular approaches for evaluating and validating the performance of machine learning (ML) models by splitting datasets into subsets. We used K-fold cross-validation, where the data was divided into K equally sized folds. The models were trained on K-folds and tested on the remaining fold. This process was repeated K times, and each fold was utilized once as the validation set. All metrics were computed based on their meaning and could be compared with the results obtained without using the cross-validation technique [53].

### 2.5. Evaluation Criteria

When implementing machine learning models, the performance of classifier models was assessed utilizing metrics that represent valuable information of the performance of algorithms. Each algorithm was evaluated by these metrics which are shown briefly in Table 2 [54].

## 3. Results

The entire experiment was conducted in the Colab environment, operating on Windows, with the hardware listed in Table 3. The experiments were implemented in Python as well.

In this study, 30 features were selected based on their relevance, as reported and summarized in Table 1. All features were used in the dataset to train the ML models as input.

The dataset, which included vocal features, was standardized because ML does not perform well when numerical values have different scales, which is why all data was standardized according to the following formula [24]:

$$z=\frac{x-\mu }{\sigma }$$

where

- *x* is the original data point;
- *µ* is the mean of the data;
- *σ* is the standard deviation of the data.

Next, the dataset was split into 80% train and 20% test subset to develop the models. The training data was useful for learning our model, which was not suitable for generalizing the model’s performance. In this research, we report on all metrics based on test models. To ensure reliability, we implemented 5-fold cross-validation to evaluate model performance. To select the best models, we used important factors mentioned earlier, including accuracy, recall or sensitivity, precision, F1-score, and ROC-AUC score. In this study we compared all metrics to select the best models for distinguishing PD from healthy individuals utilizing cross-validation.

As indicated in Table 4, the random forest (RF) model achieved the most stable and overall superior performance among all the classifiers evaluated. It attained the highest accuracy (82.7% ± 0.10) as well as the highest ROC-AUC value (0.896 ± 0.07), indicating its effectiveness in discriminating between Parkinson’s disease (PD) patients and healthy subjects (HS). Although its recall (0.7500 ± 0.15) was slightly lower than that of some other models, it still demonstrated good sensitivity in detecting PD. RF’s performance was based on the following best-set hyperparameters: n-estimators = 200 (number of trees), max-depth = 10 (maximum depth of each tree), and min-samples-split = 2 (minimum samples required to split a node).

Support vector machine (SVM) SVC(C = 10, gamma = 0.1, kernel = linear) and logistic regression (LR) (C = 10, penalty = l1, solver = liblinear) models performed similarly, with accuracies of 75.29% and ROC-AUC values of 0.826 and 0.813, respectively. Both models exhibited stable recall and F1-score values but did not outperform RF overall. Even though hyperparameters were tuned for both models, RF remained the top-performing model.

Interestingly, the naïve Bayes (NB) model (var_smoothing = 1 × 10^−9^) achieved the highest recall (0.8250 ± 0.18), making it the most sensitive in detecting PD cases. However, its lower accuracy (73.9% ± 0.15) and ROC-AUC (0.818 ± 0.13) limited its overall reliability as a standalone model. Recall is critical in medical diagnostics, as it helps minimize false negatives. Nevertheless, the relatively low precision of NB (0.7312 ± 0.16) and F1-score (0.7578 ± 0.14) suggest that while the model is highly sensitive, it is less specific.

The decision tree (DT) (max_depth = 8, min_samples_leaf = 2, min_samples_split = 4) and K-nearest neighbors (KNN) (Algorithm = brute, n_neighbors = 7) models showed the poorest results across all metrics. KNN, in particular, had the lowest precision (0.6222 ± 0.11) and ROC-AUC (0.676 ± 0.06), which limits its clinical utility.

**Figure 2:**
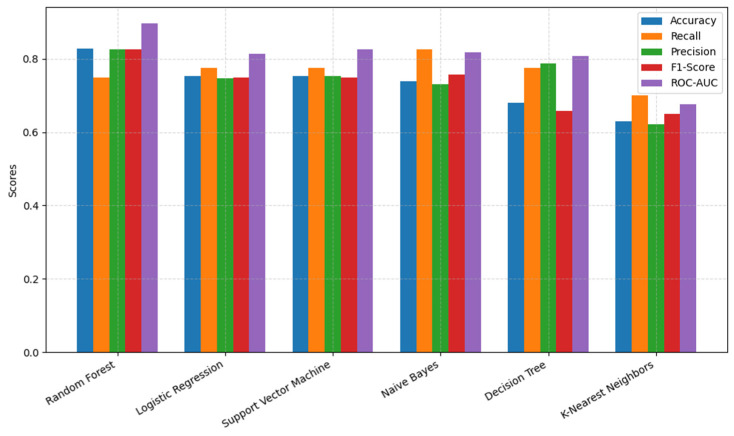
Performance of different algorithms based on metrics.

The grouped bar chart (Figure 2) illustrates the results presented in Table 4. Notably, RF demonstrated the best results in terms of ROC-AUC, accuracy, F1-score, and precision score, while KNN had the worst performance across all metrics. Recall plays a significant role in detecting PD. Although NB had the highest recall rate, SVM, LR, and DT achieved similar scores. The KNN had the lowest recall rate compared to the other models.

**Figure 3:**
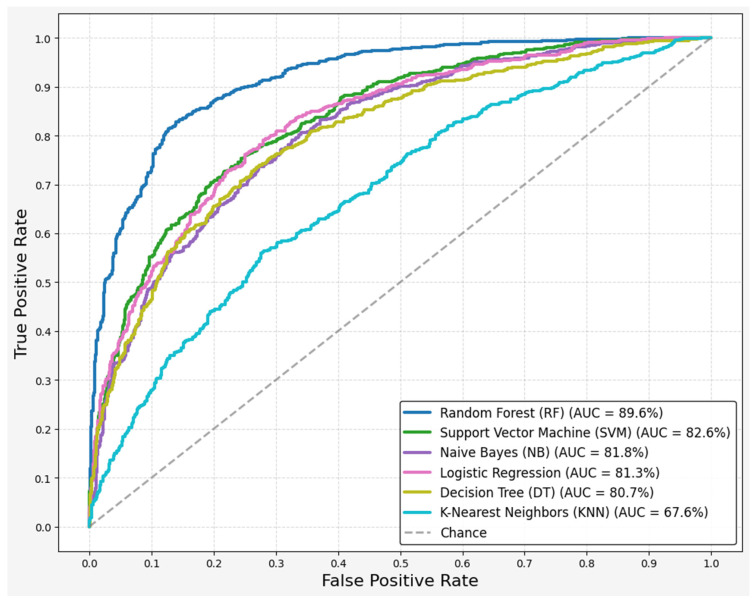
Receiver operating characteristic (ROC) curves for machine learning algorithms.

In addition to random forest, naïve Bayes, logistic regression, decision tree, K-nearest neighbors (KNN), and support vector machine (SVM) were also evaluated, as shown in Table 4. Naïve Bayes had the highest recall score, indicating a relationship between recall and PD detection from HS. However, this model suffered from lower accuracy and other important metrics. In this research, RF, SVM, and LR demonstrated the best performances across multiple metrics. These models obtained the highest rate in accuracy, precision, and F1-scores. These models showed a better balance between sensitivity and specificity. Specifically, the ROC-AUC scores for RF, SVM, and LR were 89%, 82%, and 81%, respectively, while NB and DT scored 81% and 80%, respectively (Figure 3). KNN had the lowest performance across all metrics.

Table 5 provides valuable information to compare previous studies and our outcomes. Our study adopted long-term, short-term, and non-standard features to build machine learning models. We could achieve 89.65% and 82.65% of ROC-AUC using random forest and SVM, respectively. Naïve Bayes (NB) was also approved with the highest rate of recall (82.50%) for Parkinson detection.

## 4. Discussion

Our study investigated the potential of machine learning models in distinguishing Parkinson’s disease patients from healthy individuals using vocal features. By analyzing long-term, short-term, and non-standard acoustic parameters, we aimed to develop a robust classification framework. Our findings indicate that random forest achieved the highest classification performance, followed by support vector machine, logistic regression, and naïve Bayes, suggesting that voice impairments in Parkinson’s disease can serve as reliable digital biomarkers.

Our work is aligned with prior research demonstrating the efficacy of machine learning in Parkinson’s disease detection through voice analysis. Iyer et al. employed Parselmouth, a Python interface for Praat, to extract long-term features, while we opted for Praat directly to minimize discrepancies. Unlike Iyer’s study, which focused on specific feature subsets, we incorporated additional non-standard measurements, such as pitch period entropy and recurrence period density entropy, achieving a broader representation of vocal impairments in Parkinson’s disease [39].

Through our analysis, the RF model was selected as the best option for avoiding overfitting among the tested algorithms. In contrast to the other models in this study, the RF model, based on decision trees, is well designed to model nonlinear relationships and can handle the noisy data commonly found in acoustic signals indicative of PD [55]. Within this investigation, grid search was used to select our hyperparameters. Although the selected hyperparameters did not significantly improve performance metrics for the KNN models, the results were different for the RF and SVM models [56].

Although RF and SVM were analyzed in our study, we also compared our findings with those from previous research. For instance, Iyer et al. explored random forest, logistic regression, and Convolutional Neural Networks. While our logistic regression and random forest models did not achieve the same level of performance as Iyer’s, our overall model performance was significantly higher in most cases. This contrast highlights the effectiveness of our selected feature set and machine learning approach in distinguishing Parkinson’s disease from healthy individuals [39].

In the present study, we employed 5-fold cross-validation to enhance robustness while reducing computational time. Our research demonstrated superior performance when comparing commonly used models, which are crucial for distinguishing PD from HS. In contrast, Wroge et al. did not comprehensively explore different machine learning models and utilized 10-fold cross-validation, which can increase computational time without yielding significant improvements [38].

In our approach, we combined non-standard features with long-term features and MFCC1-12 to evaluate the performance of various models (as shown in Figure 3). Our findings revealed that RF was the best model for predicting PD from HS. Notably, among all MFCC, MFCC2 emerged as the most relevant for interpreting Parkinson’s disease, as demonstrated by Tracey et al. [30]. Although we aimed to integrate all features to develop robust models, previous studies have typically analyzed features based on either non-standard or long-term features, often excluding short-term features despite their significance or disregarding non-standard measurements altogether [26].

Our findings demonstrate that random forest is particularly well-suited for handling complex, nonlinear relationships in acoustic data. Its decision tree-based structure enables effective modeling of voice impairments, which may exhibit intricate patterns that simpler classifiers struggle to capture. Additionally, support vector machine and naïve Bayes exhibited strong recall scores, highlighting their potential in prioritizing sensitivity in Parkinson’s disease screening.

Our models could assist neurologists in distinguishing PD from HS, potentially leading to earlier intervention. Early detection would allow for the timely prescription of medications, such as L-dopa, which can help improve speech impairments associated with PD, even if they do not fully restore normal speech. Detecting Parkinson’s at the prodromal stage could significantly enhance patient outcomes by enabling earlier management of symptoms [40]. 

In our investigation, the study was based on a cross-sectional study analysis of voice impairment based on ML models. A longitudinal study can be investigated as a useful approach to study any correlation between Parkinson progression and speech change. In longitudinal study, Unified Parkinson’s Disease Rating Scale (UPDRS) can provide valuable information regarding the disease progression of motor symptoms. However, voice impairment follows a different pattern from motor symptoms especially when the severity of the disease is considered over time. This indicates that separate scales should be developed to assess voice-related disease severity. According to Wright ant et al., Parkinson’s voice features were analyzed and monitored across different severity categories using one-way analysis of variance and support vector regression. Significant changes and trends were found. The study confirmed that voice could be used to detect PD at early and late stages and provide warnings about disease progression [57]. Thus, our study focuses on predicting Parkinson’s disease, and we adopted a different approach. Although they used common features, they aimed to find correlations between PD progression over time.

In our study, RF was the best model, while KNN was the worst. However, some study concluded that the AdaBoost classifier had the highest accuracy rate, while KNN was the least accurate [58].

## 5. Limitation

Our dataset comprises 81 participants (40 Parkinson’s disease (PD) patients and 41 healthy individuals), with important limitations when considering the generalization of the findings. While our dataset provides valuable information that can be used to build models to distinguish PD patients from healthy individuals, there are inherent challenges in recruiting participants for the clinical studies that this dataset supports. Recruiting both PD patients and healthy individuals for voice sampling studies can be difficult, and participants may face barriers such as mobility issues or health-related conditions, making it increasingly challenging to obtain consent. Moreover, healthy individuals may not be inclined to participate in the research, which can lead to potential bias in the participant selection process. These recruitment issues may limit the diversity and representativeness of the sample, and efforts to expand the dataset by including a variety of demographic groups are important to improve both the robustness and applicability of the findings. Apart from these challenges, the lack of participants also makes it difficult to generalize our model in medical research because of the lack of data. Furthermore, as we did not collect the data ourselves, we had limited control over the data collection process. This was another challenge in studies of this kind as greater control over potential sources of error could significantly improve the ability to optimize and build effective models.

## 6. Conclusions

This work established that voice characteristics are effective in differentiating Parkinson’s disease (PD) patients from healthy individuals using machine learning models at either early stage or prodromal phases. Since neurologists typically diagnose PD only after the onset of motor symptoms, leveraging non-motor symptoms such as speech offers a promising, non-invasive approach for early detection. In this approach, machine learning plays a crucial role by analyzing various voice features, including long-term, short-term, and non-standard parameters, which have been shown to provide strong discrimination between PD patients and healthy subjects (HS). This approach applies various machine learning algorithms, including random forest (RF), logistic regression (LR), naïve Bayes, K-nearest neighbors (KNN), and support vector machine (SVM). Our findings indicate that RF and SVM achieved the highest performance in classifying PD patients with the highest accuracy and ROC. Among all models, NB achieved the highest recall score, which is the most important metric for detecting PD from healthy subjects (HS). Further research should focus on improving classification accuracy by integrating multi-modal approaches and advanced machine learning techniques. Additionally, real-world applicability requires the validation of these models on larger and clinically diverse populations. For example, smartphones can assist neurologists in monitoring patients and even detecting Parkinson’s disease in healthy subjects. The increasing adoption of voice-based screening technologies for early PD diagnosis holds significant potential in enabling neurologists to detect PD before motor symptoms appear, facilitating earlier medical intervention and improved clinical outcomes.

## Figures and Tables

**Table 1:** List of features.

Features	Number of Features	Description
Jitter	2	Measures variability in vocal fold vibration frequency
Shimmer	5	Measures amplitude fluctuations in vocal cycles
NHR	1	Noise-to-harmonics ratio
HNR	1	Harmonics-to-noise ratio
Pitch	1	Fundamental frequency of vocal fold vibration
Intensity	1	Overall loudness of the voice
Formant	4	Resonant frequencies of the vocal tract
CPPS	1	Measures prominence of spectral peaks
PPE	1	Measures irregularity in speech pitch to distinguish between natural variations and pathological speech
RPDE	1	Measures regularity of voice signal
MFCC	12	Represents spectral envelope of the signal, useful for voice quality analysis

**Table 2:** Metrics, formulas, and descriptions.

Metrics	Formula	Description
Accuracy	$\mathrm{Accuracy}=\frac{TP+TN}{TP+TN+FP+FN}$	Proportion of correctly classified instances
Recall	$\mathrm{Recall}=\frac{TP}{TP+FN}$	Proportion of actual positives correctly identified
Precision	$\mathrm{Precision}=\frac{TP}{TP+FP}$	Proportion of predicted positives that are actually positive
F1-Score	$F1\text{-}\mathrm{Score}=2\cdot \frac{\mathrm{Precision}\cdot \mathrm{Recall}}{\mathrm{Precision}+\mathrm{Recall}}$	Harmonic means precision and recall
ROC-AUC	-	Area under the receiver operating characteristic curve.
MSE	$\mathrm{MSE}=\frac{1}{n}\sum_{i=1}^{n}{\left(y_{i}-\hat{y_{i}}\right)}^{2}$	Mean squared error between predicted and actual values

**Table 3:** System implementation environment.

Resource	Details
CPU	i5 Gen6
RAM	12.67 GB
GPU	4 GB Tesla T4, 15,360 MiB
Software	Python 3.10.12 and 3.12.8

**Table 4:** Cross Validation Results.

Algorithm	Accuracy	Recall	Precision	F1-Score	ROC-AUC
Random Forest (RF)	0.8272 ± 0.10	0.7500 ± 0.15	0.8257 ± 0.14	0.8251 ± 0.1	0.8965 ± 0.07
Logistic Regression (LR)	0.7529 ± 0.06	0.7750 ± 0.09	0.7467 ± 0.12	0.7487 ± 0.07	0.8132 ± 0.09
Support Vector Machine (SVM)	0.7529 ± 0.06	0.7750 ± 0.04	0.7529 ± 0.09	0.7487 ± 0.04	0.8263 ± 0.09
Naive Bayes (NB)	0.7397 ± 0.15	0.8250 ± 0.18	0.7312 ± 0.16	0.7578 ± 0.14	0.8181 ± 0.13
Decision Tree (DT)	0.6801 ± 0.16	0.7750 ± 0.09	0.7871 ± 0.16	0.6589 ± 0.09	0.8071 ± 0.11
K-Nearest Neighbors (KNN)	0.6301 ± 0.1	0.7000 ± 0.15	0.6222 ± 0.11	0.6493 ± 0.09	0.6760 ± 0.06

**Table 5:** Comparison of previous ML-based voice analysis studies for Parkinson’s disease and our study.

Study	Featured Used	Machine Learning Models	Best Performance
Our research	Long-term features, short-term features, PPE, RPDE	RF, SVM, NB	89.65% (ROC-AUC), 82.63% (ROC-AUC) 82.50% (Recall)
Fred Prior [41]	Long term and short-term features	RF, LR, CNN	78% (AUC), 78% (AUC), 97% (AUC)
Max little [26]	Long-term features, non-standard measurement	SVM	90.4% (accuracy)
Wroge [38]	GeMaps features	Gradient Boosted Decision Tree	82% (accuracy), 65% (recall)

## Data Availability

The data applied to this study to assess systems performance were obtained from the figshare repository: Parkinsons dataset, which is publicly available at: https://figshare.com/articles/dataset/Voice_Samples_for_Patients_with_Parkinson_s_Disease_and_Healthy_Controls/23849127 (accessed on 4 August 2023).

## Abbreviations

The following abbreviations are used in this manuscript:

PD	Parkinson’s disease
HS	Directory of open access journals
CPPS	Smoothed cepstral peak prominence
PPE	Pitch Period Entropy
RPDE	Recurrence period density entropy
MFCCs	Mel-frequency cepstral coefficients
RF	Random Forest
KNN	K-nearest neighbors
ML	Machine Learning
NB	Naïve Bayes
SVM	Support vector machines
LR	Logistic Regression
ROC	Receiver-operating characteristic curve
AUC	Area under the curve
RBD	REM sleep behavior disorder
REM	Rapid Eye Movement
Eq	equation
TP	True Positive
FP	False Positive
TN	True Negative
FN	False Negative
MSE	Mean Squared Error
CV	Cross Validation
UPDRS	Unified Parkinson’s Disease Rating Scale
PIGD	Postural Instability and Gait Disorders
CPP	Cepstral Peak Prominence
